# Microbial Community Structure–Function Relationships in Yaquina Bay Estuary Reveal Spatially Distinct Carbon and Nitrogen Cycling Capacities

**DOI:** 10.3389/fmicb.2018.01282

**Published:** 2018-06-14

**Authors:** Brandon Kieft, Zhou Li, Samuel Bryson, Byron C. Crump, Robert Hettich, Chongle Pan, Xavier Mayali, Ryan S. Mueller

**Affiliations:** ^1^Department of Microbiology, Oregon State University, Corvallis, OR, United States; ^2^Oak Ridge National Laboratory, Oak Ridge, TN, United States; ^3^Graduate School of Genome Science and Technology, The University of Tennessee, Knoxville, Knoxville, TN, United States; ^4^Department of Civil and Environmental Engineering, The University of Washington, Seattle, WA, United States; ^5^College of Earth, Ocean, and Atmospheric Sciences, Oregon State University, Corvallis, OR, United States; ^6^Lawrence Livermore National Laboratory, U.S. Department of Energy, Livermore, CA, United States

**Keywords:** metagenomics, metaproteomics, estuary, biogeochemical cycling, Yaquina Bay, free-living, particle-attached

## Abstract

Linking microbial community structure to ecological processes requires understanding of the functional roles among individual populations and the factors that influence their distributions. These structure–function relationships are particularly difficult to disentangle in estuaries, due to highly variable physico-chemical conditions. Yet, examining microbe-mediated turnover of resources in these “bioreactor” ecosystems is critical for understanding estuarine ecology. In this study, a combined metagenomics and metaproteomics approach was used to show that the unequal distribution of microbial populations across the Yaquina Bay estuary led to a habitat-specific taxonomic and functional structure and a clear spatial distribution in microbe-mediated capacities for cycling of carbon and nitrogen. For example, size-fractionation revealed that communities inhabiting suspended particulate material encoded more diverse types of metabolisms (e.g., fermentation and denitrification) than those with a planktonic lifestyle, suggesting that the metabolic reactions can differ between size fractions of the same parcel of an estuarine water column. Similarly, communities inhabiting oligotrophic conditions in the lower estuary were enriched in genes involved in central carbon metabolism (e.g., TCA cycle), while communities in the upper estuary were enriched in genes typical of copiotrophic populations (e.g., cell growth, cell division). Integrating gene and protein data revealed that abundant populations of Flavobacteriales and Rhodobacterales encoded similar genomic functions, yet differed significantly in protein expression, dedicating a large proportion of their respective proteomes to rapid growth and division versus metabolic versatility and resource acquisition. This suggested potentially distinct life-strategies between these two co-occurring lineages and was concomitant with differing patterns of positive evolutionary selection on their encoded genes. Microbial communities and their functions across Yaquina Bay appear to be structured by population-level habitat preferences, resulting in spatially distinct elemental cycling, while within each community, forces such as competitive exclusion and evolutionary selection influence species life-strategies and may help maintain microbial diversity.

## Introduction

Estuaries host communities of microorganisms that influence the exchange of nutrients between terrestrial, freshwater, and marine biomes (Cole and Caraco, 2001; Bauer et al., 2013). These boundary ecosystems often exhibit strong spatiotemporal gradients in salinity, turbidity, and resource availability, resulting in microbial communities with highly variable characteristics across these conditions (Crump et al., 2004; Jeffries et al., 2016). Biotic and abiotic dynamism is particularly evident in estuaries during winter along the Oregon coast when heavy overland precipitation and steep nearshore topography cause coastal river flooding events that transport significant amounts of suspended particulate matter into estuarine water columns (Hickey and Banas, 2003; Hastings et al., 2012; Goñi et al., 2013).

Microbes colonizing such particles represent an important component of aquatic biogeochemical cycles by acting to liberate particulate carbon and nutrients to the planktonic microbial loop through decomposition processes, and by supplementing higher trophic levels of the food web through resulting biomass production (Goulder, 1977; Wainright, 1990; Brown and Ozretich, 2009; Stocker, 2012). Studies examining microbial communities in Oregon estuaries have observed significant differences in the phylogenetic composition and respiratory activity between particle-associated and free-living microbial communities and across marine–estuarine–riverine gradients (Crump et al., 1998, 1999, 2017; Smith et al., 2013). Beyond phylogenetic characterization, however, the functional roles of microbial communities and their relative influence on ecological processes occurring in different habitats within heterogeneous estuarine systems have seldom been examined (Simon et al., 2014).

Applying a combination of high-throughput DNA sequencing and metaproteomics techniques, we investigated the phylogenetic and functional structure of microbial communities with two contrasting, operationally defined lifestyles (particle-associated and free-living) in two spatially separated locations (upper and lower) of Yaquina Bay, Oregon, United States. Using these data, we estimated how the mechanisms of microbe-mediated carbon and nitrogen turnover differed between the four habitats. This was achieved using previously identified metabolic marker genes and proteins, whose relative abundances provide a proxy for different components of aquatic microbe-mediated biogeochemical cycles (Lauro et al., 2011; Llorens-Marès et al., 2015). We hypothesized that habitat preference (i.e., a distribution biased toward a specific habitat) at the population level would lead to communities with distinct taxonomic and functional structures, corresponding with habitat-specific carbon and nitrogen cycling on both large (kilometers, between locations) and small (microns, between particle-attached and free-living) spatial scales (Hyndes et al., 2014) in the Yaquina Bay estuary.

To understand the ecology of successful microorganisms in this ecosystem, we also reconstructed the encoded genomes and expressed proteomes at the time of sampling from 15 populations in two dominant lineages (Flavobacteriales and Rhodobacterales) to determine whether their life-strategy – i.e., the set of traits used for survival, growth, and reproduction (Barnard et al., 2013) – contributed to their pervasiveness across habitats. To determine if life-strategy of the two taxa was consequential for their coexistence and codominance (i.e., whether they displayed overlapping or divergent life-strategies and, thus, occupied a similar or different niche space), we compared the relative abundances of their expressed proteins and their protein-coding genes. Similarly, we examined positive evolutionary selection in these two lineages using classical analyses of synonymous and non-synonymous mutation rates on protein-coding genes to determine whether evolutionary pressure on each lineage was related to its life-strategy.

## Materials and Methods

### Sample Collection and Processing

Surface water samples were collected on November 22, 2014 from two sites in Yaquina Bay (OR, United States). Samples were taken from the top 1 m of the water column at a tidal height of ∼1.8 m near slack current (-0.1 m/s) in both the riverine end-member of the estuary (hereafter, “upper”: 44.58°N, -123.99°W) and the coastal estuary mouth (hereafter, “lower”: 44.62°N, -124.04°W), which are separated by 8 km (Supplementary Figure S1). Along with tidal influence, Yaquina River flow was ∼17 m^3^/s (Oregon Water Resources Department, Station ID 14306030), resulting in upper and lower estuary sampling sites that represent differing salinity and nutrient zones during our sampling period (Brown and Ozretich, 2009; Shafer et al., 2016). Triplicate 12 L water samples were collected in acid-washed carboys and filtered through in-line 3-μm and 0.22-μm PES membranes (Pall Corporation, Ann Arbor, MI, United States) to recover operationally defined particle-associated and free-living microbial communities, respectively. Collected biomass was stored at -80°C until processing.

Community DNA was extracted using the CTAB protocol described in (Doherty et al., 2017). Total protein was extracted using SDS lysis method detailed in (Bryson et al., 2016, 2017). Extractions from 1 and 11 L of water yielded sufficient quantities of analytes for further analyses (1–5 μg DNA and 150–300 μg of protein, respectively).

### 16S Amplicon Library Construction and Analysis

The v4 locus of the 16S rRNA gene was amplified and sequenced on the Illumina MiSeq (Illumina, Inc., San Diego, CA, United States) using the two-step PCR protocol of the Nextera XT index kit. First-step PCRs were performed with 30 cycles using ∼50 ng of DNA, AccuStart II PCR ToughMix Polymerase (QuantaBio, Beverly, MA, United States) following manufacturer instructions, and primers encoding universal prokaryotic v4-complement sequences (515F and 806R) (Takahashi et al., 2014). AMPure XP beads were used for product purification. Second-step PCRs were performed per Nextera XT kit instructions.

Libraries were normalized by concentration, pooled, and sequenced using an Illumina MiSeq v2 kit (251 bp, paired-end reads), generating >17,000 reads per library. Reads were trimmed and quality filtered using FastQC and Sickle (Joshi and Fass, 2011). Low quality (<25 phred scores across 15 base sliding windows) sequences within reads were trimmed and >200 bp reads were retained. The “mothur” program was used to remove adapter regions and for read-pair assembly, unsupervised OTU clustering (97% identity), taxonomic assignment with the RDP classifier, rarefaction, relative abundance calculations, and OTU table generation (Schloss et al., 2009).

### Metagenome Sequencing, Assembly, and Annotation

Metagenome libraries were prepared using Nextera XT (Illumina, Inc., San Diego, CA, United States) and Wafergen (Wafergen Bio-systems, Fremont, CA, United States) kits. Libraries were quantified with Bioanalyzer HS-DNA Chips, normalized by concentration, and pooled. Metagenome sequencing was performed on Illumina HiSeq 3000 using 151 bp long, paired-end reads and yielded >16 Gb per library (>32 M reads). Metagenome reads were filtered as defined for 16S amplicon reads, with the exception that the length threshold was 100 bases. IDBA-UD (Peng et al., 2013) was used for *de novo* metagenome assembly of the combined read sets from all three replicate community DNA samples in each of the four habitats (i.e., upper and lower estuary communities with both particle-associated and free-living lifestyles). All 16S-amplicon and metagenome sequences are available from NCBI (BioSample SAMN04917373, BioProject PRJNA320136, short read archive SRS1422236 with accession numbers SRX1738750-90 for 16S-amplicon reads, and SRX1738728-32 for metagenome reads).

Predicted coding sequences (CDS) on contigs were determined using Prodigal (Hyatt et al., 2012). Taxonomic assignment of CDS was based on best hits to the RefSeq database (downloaded June 14, 2014) using the Diamond algorithm in “blastp” mode (Buchfink et al., 2015). Diamond blastp was chosen for annotation to the Class taxonomic level based on speed and sensitivity tests comparing the algorithm with traditional blastp and Diamond blastp in sensitive mode (Supplementary Figure S2). The GhostKoala server was used to functionally annotate each CDS by assigning a KEGG Orthology (KO) number (Kanehisa et al., 2016). Additional functional assignments of CDS were based on best rpsBLAST hits to protein families from the Pfam (Finn et al., 2016) and cluster of orthologous genes (COG) (Huerta-Cepas et al., 2016) databases.

Read depths of contigs/scaffolds and of CDS were defined by separately aligning reads from all libraries against each of the four resulting assembles using the Bowtie2 program (Langmead and Salzberg, 2012). The relative abundances of CDS were approximated using the reads per kilobase of genome equivalents (RPKG) metric, which was calculated with “MicrobeCensus.” This metric uses CDS read-depth and a genome equivalent metric based on metagenome library size and single-copy marker genes in order to normalize relative abundances of CDS across metagenomes (Nayfach and Pollard, 2015).

Contigs from each metagenome were automatically binned into organism-specific sets using the “maxbin2.1” program, which uses contig read-depths and tetranucleotide (TN) frequency data for bin assignment (Wu et al., 2016). More than 600 total bins were auto-generated. The lineage-specific workflow of “CheckM” (Parks et al., 2015) was used to assess initial bin quality by calculating completeness and contamination metrics. Bins with >85% completeness (83 bins; bolded typeface in Supplementary Table S1) were manually curated using “Vizbin” (Laczny et al., 2015) and “mmgenome” (Karst et al., 2016). Outlying contigs were identified in plots of GC content, TN frequency, and read-depths and removed. Resulting bins were reassessed in “CheckM,” and those with >85% completeness and <10% contamination were analyzed further (40 bins; bolded and italicized typeface in Supplementary Table S1). Taxonomy of manually curated bins was determined by BLAST-based annotation of binned CDS, by comparison to the “amphora2” marker gene set (Wu and Scott, 2012), and with “CheckM” rerun with the taxon-specific workflow. Thirty-nine bins with >85% completeness and <10% contamination were retained for further population-level analyses.

### Peptide Mass Spectrometry, Database Construction, and Protein Identification

Extracted proteins were digested with trypsin at room temperature (enzyme:substrate ratio of 1:100 w:w). Twenty-five micrograms of peptides were analyzed for each of the 12 metaproteomes using 2D-Liquid Chromatography Tandem Mass Spectrometry (LC-MS/MS) with previously described conditions (Bryson et al., 2016). LC-MS/MS measurements were performed on an LTQ Orbitrap Elite mass spectrometer (Thermo Scientific, Waltham, MA, United States) (Washburn et al., 2001). MS and MS/MS scans were acquired with a resolution of 30,000 and 15,000, respectively, and the 10 most abundant precursor ions were selected for MS/MS analysis by higher-energy collisional dissociation.

Sipros Ensemble (Guo et al., 2017) was used to search all MS spectra against a peptide database constructed from all metagenome CDS clustered at 100% identity using cd-hit (Li and Godzik, 2006), and with reverse sequences of all peptides added as decoys to calculate false discovery rate (FDR) (Bryson et al., 2016). Searches considered 7–60 residue peptides with a maximum of two missed tryptic cuts. The two-peptide rule – one unique plus one shared peptide, or two unique peptides – was used to determine confident protein identifications, and a 1% FDR at the peptide level was implemented as in (Bryson et al., 2016). Normalized balanced spectral counts (NBSC) of peptides for identified proteins were used as measures of protein relative abundances across all samples (Supplementary Table S2). The proteomics data have been deposited to the ProteomeXchange Consortium via the PRIDE (Vizcaíno et al., 2016) partner repository with the dataset identifier PXD008093.

### DNA Sequence Analyses

The relative abundances of KO, Pfam, and COG protein families within each metagenome were approximated based on the cumulative RPKG of all CDS assigned to a given protein family. The relative abundance of COG categories was calculated by summing RPKG for all CDS assigned to COG numbers in each COG category.

To infer carbon and nitrogen cycling potential of each microbial community, relative abundances of metabolic marker genes (KO numbers) defined in previous studies (Lauro et al., 2011; Llorens-Marès et al., 2015; Vavourakis et al., 2016) were calculated using marker KO RPKG ratios. A total of 40 marker genes were used in the analysis, representing 10 microbe-mediated elemental cycling reactions (Supplementary Table S3). Metabolic potential for each metagenome was calculated as in (Lauro et al., 2011) and averaged across replicates. The relative abundance of proteins (in NBSC) encoded by marker KO genes were similarly analyzed. Statistically significant differences in the relative abundances of element cycling processes between habitats were assessed using ANOVA with the Tukey–Kramer *post hoc* test and Benjamin-Hochberg FDR controlled at 5% using the “STAMP” software package (Parks et al., 2014).

OTU relative abundances based on 16S amplicon sequencing data were summed at Order-level taxonomic groupings for community diversity analyses. Biplots of principal component analyses (PCA) examining the beta-diversity of communities were produced using the “pca3d” library in R (Weiner, 2017). Weighted Unifrac calculations, PCA, Mantel tests, Procrustes analyses, and permutational ANOVA tests were performed in R using the “vegan” package (Oksanen et al., 2017). Faith’s (1992) phylogenetic alpha-diversity was calculated using the “picante” package and significant differences between samples were defined using pairwise Welch’s *t*-tests. R scripts and input data for statistical calculations can be found in Supplementary Table S2.

Significantly discriminating taxonomic and functional features between communities were identified using the linear discriminant analysis (LDA) method implemented in “LEfSe” (Segata et al., 2011). “Enrichment” of a feature refers to one with an LDA score >2.0 (log 10) using the strict all-against-all multi-class analysis and a *p* < 0.05 cutoff for the among- and between-class LEfSe tests. LEfSe, by default, performs significance testing on normalized relative abundances. Thus, a correction variable was added to input files when the feature abundance was in RPKG, which is based on a unique genome equivalent metric calculated for each sample (Nayfach, personal communication).

### Positive Selection Detection on Groups of Orthologous Proteins

ProteinOrtho was used to predict orthologous protein groups (OGs) shared between populations (Lechner et al., 2011). OGs were scanned for evidence of horizontal transfer using GENECONV, and those with significant hits were not considered in further analyses (Sawyer, 1989; Posada and Crandall, 2001). The “ETE3” toolkit (Huerta-Cepas et al., 2016) was used to discover evidence of positive selection on OGs using Codeml and PAML. Clustal Omega with trimAL and RaxML were implemented for multiple codon sequence alignments and tree-building, respectively (Sievers et al., 2011; Stamatakis, 2014). Trees were visualized with the Interactive Tree of Life server (Letunic and Bork, 2016).

Models M1 vs. M2, M7 vs. M8, and M8 vs. M8a were used as sets of null and alternative models, respectively, for defining a threshold of significance for positive selection (Yang et al., 2000). A significant difference in dN/dS ratio (omega) based on likelihood ratio tests (LRT) of model outcomes with the BEB posterior probability method was used to infer the best model fit for a given OG alignment (Yang et al., 2005). LRT *p*-values from all tests were corrected by controlling at a FDR of 5%. If an OG had a corrected *q*-value < 0.05 under any of the three models, this gene group was considered to be under putative positive selection (e.g., as in Tang and Zhang, 2007).

## Results

### Community Taxonomic Structure and Diversity

**FIGURE 1:**
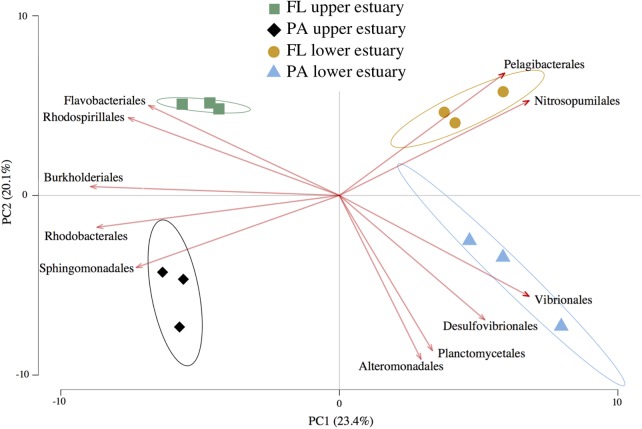
Euclidean distance PCA with feature biplots of taxonomic community structure. Plotted data are relative abundance of 16S OTUs grouped at Order-level taxa. Sample triplicates per habitat (points) are plotted alongside a set of the most important Orders driving sample separation (vectors). Arrow direction and magnitude represent both the covariance between features (Orders) and their effect on PC loadings. Ellipses represent 95% confidence intervals for centroid positions. FL, free-living; PA, particle-attached.

Amplicon sequencing of 16S v4 loci and clustering at 97% identity yielded 6,792 OTUs from ∼240,000 total paired-end reads (>17,000 paired-end reads per replicate) across all four sampled habitat communities (Supplementary Table S4). The OTU-level Unifrac distance matrix and Order-level Euclidean distance matrix calculated from OTU counts yielded highly similar community beta-diversity patterns (Mantel’s test, Pearson *r* = 0.962, *p* < 0.001), and PCA likewise resulted in similar data shape between these two metrics (Procrustes symmetric correlation = 0.982, *p* < 0.001). Therefore, community structure analysis was conducted using Euclidean PCA and biplotting of Order-level taxa relative abundances across samples to determine which groups most strongly drove differences in community structure (**Figure 1**). PCA indicated that 23.4% of community structure variance was explained by sampling location and 20.1% by lifestyle (**Figure 1**). However, only location significantly differentiated overall sample grouping, while lifestyle did not (Permutational ANOVA, perms = 999; df = 11; location: *p* = 0.002, *R*^2^ = 0.54; lifestyle: *p* = 0.14, *R*^2^ = 0.85). In contrast, community alpha-diversity was not different between the upper and lower estuary communities (*p* = 0.45). Rather, communities in the particle-associated lifestyle were significantly more diverse than their free-living counterparts both overall (*t*-test, *p* < 0.0001), and within each location (each *p* < 0.01; Supplementary Figure S3).

### Population-Level Habitat Preferences

Of the 6,792 OTUs recovered, 157 (2.3%) were overrepresented in one of the four estuary habitats based on significant relative abundance changes across samples using linear discriminant analysis (LDA score >2). Despite being a minor subset of all OTUs, these populations represented 75.5% of all sampled 16S reads, indicating that relatively high-abundance populations exhibited habitat preference (Supplementary Table S5).

The three most abundant Orders in all samples were Pelagibacterales, Flavobacteriales, and Rhodobacterales, collectively representing 39–54% of total 16S reads (Supplementary Figure S4). The relative habitat preferences of several populations in these three lineages influenced divergent habitat taxonomic structures (**Figure 1**). For example, the SAR11 clade member HTCC1062 (Pelagibacterales) was cumulatively the most abundant Yaquina Bay organism (OTUs 1, 14, 55) and exhibited clear habitat preference for the free-living fraction of the marine-influenced lower estuary (each OTU LDA score >3.2; Supplementary Table S5). In the Flavobacteriales and Rhodobacterales lineages, notable populations with significant habitat preference included a Cellulophaga (OTU 2; Flavobacteriales) in the free-living lifestyle of the upper estuary (LDA = 4.8) and a Thalassobius (OTU 4; Rhodobacterales) in the particulate-associated lifestyle of the upper estuary (LDA = 4.5).

An archaeal population of Nitrosopumilales (OTU 30), which represented >98% of estuary Thaumarchaeota, was strongly associated with the free-living fraction of the lower estuary (LDA score = 3.6), causing this group to be as influential as Pelagibacterales in driving unique taxonomic structure of the habitat despite its lower relative abundance (**Figure 1**). Likewise, OTUs belonging to lineages that typically inhabit anaerobic or microaerophilic environments (e.g., Desulfovibrionales) were relatively rare community members (cumulatively 0.5–2.5%; Supplementary Figure S5), but their enrichment in the particle-associated habitats drove distinct alpha-diversity (Supplementary Figure S4) and beta-diversity structure (**Figure 1**) of this lifestyle in each estuary location.

### Community Functional Structure

**FIGURE 2:**
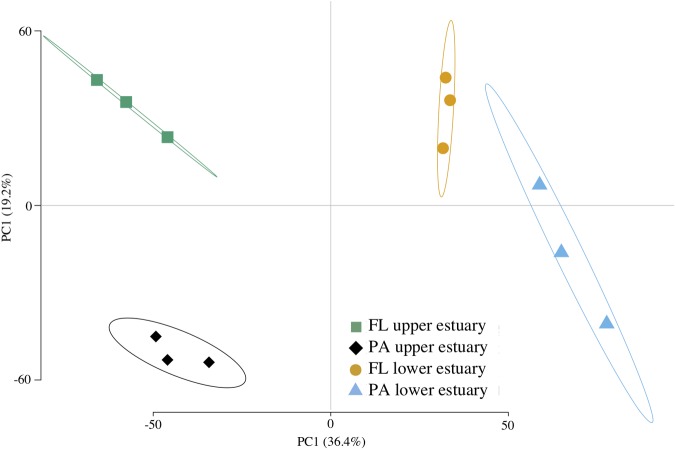
Euclidean distance PCA of functional community structure. Plotted data are KO RPKG relative abundance across samples based on metagenome CDS annotation. Ellipses represent 95% confidence intervals for centroid positions. FL, free-living; PA, particle-attached.

Assembly of ∼62 Gbp shotgun metagenome DNA sequence yielded ∼1 M contigs (1.8 Gbp) and ∼2.5 M predicted CDS across all four sampled habitats (Supplementary Table S4). Between 71 and 78% of CDS were assigned to a taxonomic group and between 32 and 58% were annotated to COG, KEGG, or Pfam protein families. Functional structure based on KEGG-annotated CDS relative abundance was significantly correlated to taxonomic structure inferred from 16S analyses (Mantel’s test, Pearson *r* = 0.832; *p* < 0.0001), leading to the similar ordination pattern (**Figures 1**, **2**).

Of the 4,764 KEGG protein families identified across all metagenomes, 432 (9.1%) were biased in their distribution between samples (Supplementary Table S6). Several functional pathways showed clear patterns of habitat enrichment based on the distribution of KEGG families (**Figure 2** and Supplementary Figure S6), including photosynthesis in the particulate-associated fractions (Supplementary Figure S6A), ABC transport in the free-living fractions, flagellar motility in the upper estuary (Supplementary Figure S6C), TCA cycle and glycolysis in the lower estuary (Supplementary Figures S6D,E), and ribosomal and transcription in the upper estuary (Supplementary Figures S6F,G).

### Community Carbon and Nitrogen Cycling Capacities

**FIGURE 3:**
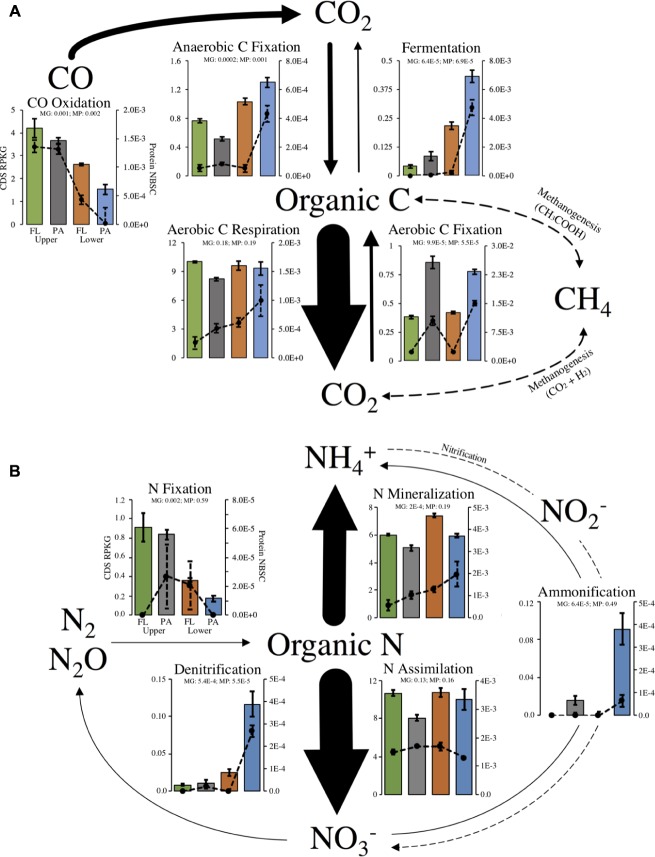
Major processes within the **(A)** C- or **(B)** N-cycles and relative abundance of biological marker genes that catalyze fluxes. Arrow sizes show relative abundance of total marker gene RPKG for each process. Bar plots show marker genes (left-axis) and lines plots show marker proteins (right-axis). Dotted arrows represent a process with no recovered marker genes or proteins. Axis labels and scales are equivalent for all plots and only drawn on one plot for clarity. Significantly non-equal potential across habitats was determined with a FDR-corrected ANOVA: *q* < 0.05). Error bars represent standard error of the mean for each set of replicate samples. MP, metaproteome; MG, metagenome in ANOVA results.

Out of 40 marker genes for 13 biogeochemical flux processes, 31 genes and 10 processes were identified in our metagenome assemblies (Supplementary Table S3). Most missing genes were involved in processes not expected to occur at detectable levels in aerobic estuarine surface water (anammox and methanogenesis). Of the 31 metagenome-encoded marker genes, 21 proteins were identified in metaproteomes (Supplementary Table S3). The relative abundance of marker genes and marker proteins within each habitat were correlated, indicating that the gene abundance for C- and N-cycling processes was generally a useful indicator of protein abundance within our samples (**Figure 3**).

As expected, the encoded genetic capacity (metagenome) for aerobic C respiration is likely the dominant pathway of estuary C flux and was equal across all habitats (FDR-corrected ANOVA, *q* = 0.18; **Figure 3A**). Aerobic C fixation potential, on the other hand, was significantly higher in the particle-associated fractions of both locations, and marker gene relative abundance for anaerobic C fixation and fermentation were overrepresented in particle-attached community of the lower estuary (**Figure 3A**). Notably, carbon monoxide (CO) oxidation genes were highly enriched in upper estuary samples, and over 68% of CO oxidation capacity was annotated to the Roseobacter clade (e.g., Roseovarius, Ruegeria).

With regard to N-cycling processes, the encoded capacity for N assimilation is likely the dominant pathway of estuary N flux and was equal across all habitats (FDR-corrected ANOVA, *q* = 0.13; **Figure 3B**). Importantly, though, encoded community capacity for N mineralization appeared to be highest in the particle-attached lifestyles in each location. Marker gene and marker protein relative abundances suggested enrichment in ammonification and denitrification potential in the marine-influenced lower estuary, particularly in the particle-associated communities (**Figure 3B**). On the other hand, marker gene abundance for N fixation was significantly enriched in the upper estuary, though detected proteins did not show this trend.

A comparison of the taxonomic distribution of 16S rRNA reads, metagenome CDS, and the N or C marker genes showed that many taxa appear to contribute disproportionately to community biogeochemical cycling capacity compared to their relative abundance in the community (Supplementary Figure S7). These taxa and processes included ammonification in Epsilon-proteobacteria (ammonification: 85%, 16S: 0.4%), fermentation in Aquificae and Planctomycetia (fermentation: 63%, 16S: 0.02%; fermentation: 9%, 16S: 0.7%), N fixation in Alpha-proteobacteria (N fixation: 81.3%, 16S: 15.3%), and aerobic C fixation in Eukaryota (C fixation: 10%, 16S: 1.4%).

### Population-Level Life-Strategies

**FIGURE 4:**
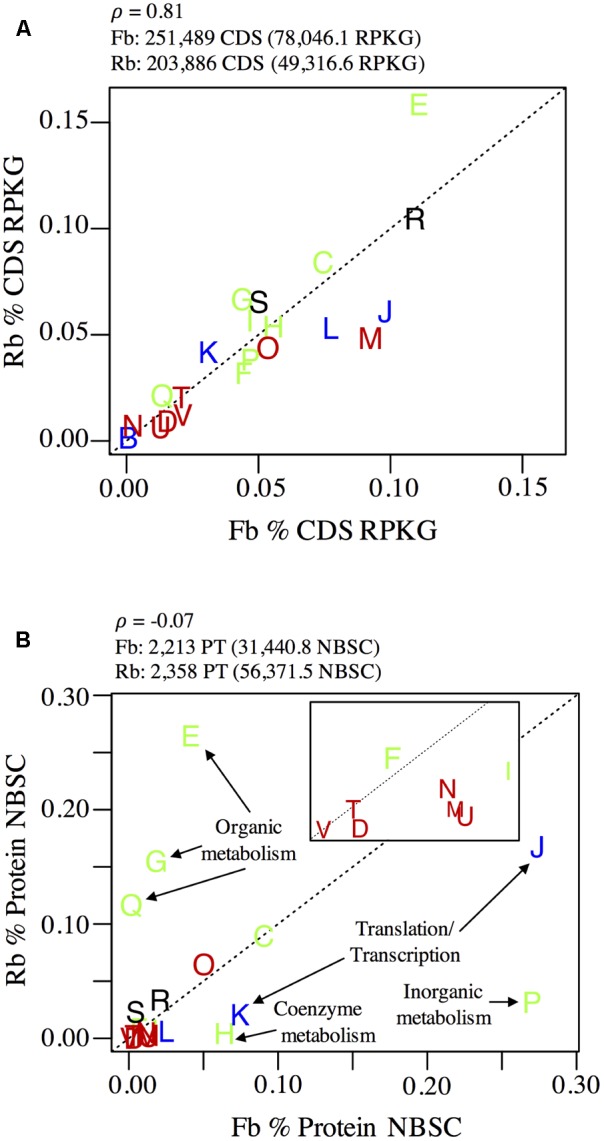
Comparison of **(A)** pangenome and **(B)** panproteome content of Rhodobacterales (Rb) and Flavobacteriales (Fb). Plots show cumulative relative abundance of lineage-specific **(A)** CDS and **(B)** proteins annotated to COG numbers and grouped at COG category level (represented by letters as points on each plot). Input and results of correlation analysis from genomic and proteomic data are shown above each plot. The inset in **(B)** is an expansion of the origin region (0–0.02 on *x*-axis and 0–0.015 on the *y*-axis). COG categories are colored according to their classification: blue, information storage and processing; green, metabolism; red, cellular processes and signaling; black, unclassified. Dotted lines indicate a 1:1 relationship. COG categories shown but not labeled are: D, cell cycle control, cell division, chromosome partitioning; M, cell wall/membrane/envelope biogenesis; N, cell motility; O, post-translational modification, protein turnover, and chaperones; T, signal transduction mechanisms; U, intracellular trafficking, secretion, and vesicular transport; V, defense mechanisms; B, chromatin structure and dynamics; L, replication, recombination and repair; C, energy production and conversion; F, nucleotide transport and metabolism; I, lipid transport and metabolism; R, general function prediction only; S, function unknown.

Flavobacteriales and Rhodobacterales were ubiquitous, though unevenly distributed (**Figure 1**), across all samples; their combined pangenome (>450,000 CDS) and panproteome (>4,500 proteins) represented 24.7 and 31.4% of total sampled genes and proteins, respectively (**Figure 4**). Fifteen well-curated population Flavobacteriales and Rhodobacterales genomes were defined from the metagenome contig binning and curation process, while bins from the other relatively abundant taxon, Pelagibacterales, could not be easily resolved (data not shown). This was reflected in 16S v4 diversity; of OTUs over 0.1% relative abundance, three were in Pelagibacterales, while 18 were Flavobacteriales and 10 were Rhodobacterales. The robust dataset of genes, proteins, and curated genomes made Flavobacteriales and Rhodobacterales lineages good candidates to test whether co-dominance across the estuary was corollary to divergent strategies for growth and resource acquisition, which is a hypothesis that has been explored for co-dominant microbial populations in other ecosystems (e.g., Violle et al., 2011).

While the Flavobacteriales and Rhodobacterales pangenomes, as determined by the relative abundance of all metagenome CDS annotated to each group, were correlated at the conservative functional classification level of COG category (Pearson’s ρ = 0.81), their panproteomes, as determined by the relative abundance of all proteins annotated to each group, were weakly correlated at this broad resolution (ρ = -0.07), indicating that proteins of different functions were being expressed by each group during sampling (**Figure 4**).

The panproteome of Rhodobacterales populations was relatively enriched for metabolism functions, with over 50% of proteins annotated to amino acid (E), carbohydrate (G), nucleotide (F), and secondary metabolite (Q) transport and metabolism COG categories (**Figure 4**). In contrast, Flavobacteriales populations devoted just 6.8% of proteins to these four COG categories and were instead enriched in translation (J, 27.4%) and transcription (K, 16.7%) functions relative to Rhodobacterales (7.4 and 2.1%, respectively). Flavobacteriales inorganic ion transport and metabolism proteins was also highly disproportionate (P, 27.0%) relative to Rhodobacterales (3.1%), though the majority of these proteins (>93%, predominately SusC and SusD) are involved in the Bacteroidetes starch utilization system (Reintjes et al., 2017).

### Evolutionary Pressure on Life-Strategies

The 10 Flavobacteriales and five Rhodobacterales population genomes recovered by metagenome contig binning had an average completeness of 93.84% and an average contamination of 2.68% (Supplementary Table S7). In total, 73 and 138 single-copy, orthologous gene groups (OGs) were shared in the Flavobacteriales and Rhodobacterales populations, respectively. A concatenated amino acid alignment of these OGs was used to estimate the phylogenetic relationship between populations in each lineage (Supplementary Figure S8), and positive selection on OGs was determined based on the classic dN/dS substitution ratio (Yang et al., 2000). Positive selection was observed in seven (9.6%) Flavobacteriales and 10 (7.2%) Rhodobacterales OGs (**Table 1**). Model statistics are reported in Supplementary Tables S8, S9. Similar rates have been reported in other non-pathogenic, or host-dependent, microbial populations (e.g., 4.5% in Nandi et al., 2010; 9% in Bulgarelli et al., 2015).

**Table 1:** Genes in the Rhodobacterales and Flavobacteriales lineages that are under putative positive selection.

OG	*p*-value	*q*-value	COG	Gene description	COG category	COG category description
Rhodobacterales						
469	0.00000	0.00000	COG2710	*chlN* [EC:1.3.7.7]	**C**	Energy production and conversion
566	0.00000	0.00000	COG0078	*argF* [EC:2.1.3.3]	**E**	Amino acid metabolism and transport
623	0.00060	0.01385	COG0765	*aapM*	**E**	Amino acid metabolism and transport
29	0.00103	0.01765	COG2379	*gckA* [EC:2.7.1.165]	**G**	Carbohydrate metabolism and transport
113	0.00115	0.01765	COG2239	*mgtE*	**P**	Inorganic ion transport and metabolism
859	0.00172	0.02372	COG0704	*phoU*	**P**	Inorganic ion transport and metabolism
282	0.00007	0.00179	COG1054	-	R	General functional prediction only
313	0.00104	0.01765	COG0824	*ybgC* [EC:3.1.2.-]	R	General functional prediction only
647	0.00000	0.00000	COG2366	[EC:3.5.1.11]	R	General functional prediction only
633	0.00000	0.00000	COG2204	*glnG*	*T*	Signal transduction
Flavobacteriales						
144	0.00214	0.02233	COG0074	*sucD* [EC:6.2.1.5]	**C**	Energy production and conversion
56	0.00007	0.00168	COG0015	*purB* [EC:4.3.2.2]	**F**	Nucleotide metabolism and transport
105	0.00037	0.00677	COG0124	*hisS* [EC:6.1.1.21]	J	Translation
123	0.00069	0.01007	COG0353	*recR*	L	Replication and repair
121	0.00000	0.00007	COG0768	*ftsI* [EC:3.4.16.4]	*M*	Cell wall/membrane/envelop biogenesis
81	0.00107	0.01299	COG2825	*hlpA*	*M*	Cell wall/membrane/envelop biogenesis
147	0.00000	0.00000	COG0386	*gpx* [EC:1.11.1.9]	*O*	Protein turnover, chaperones


*OG, orthologous group, where the three-digit numbers represent clusters of orthologs predicted by ProteinOrtho. *p*-value, *p*-value from modeling. *q*-value, Benjamini–Hochberg correction of *p*-values with FDR cut-off set at 0.05. In the COG category column, typefaces indicate the following classes: bold, metabolism; italic, cell processing and signaling; underlined, information storage and processing; normal, poorly characterized.*

In Rhodobacterales, over half of genes under selection were related to resource transport and metabolism (**Table 1**). The positively selected genes *glnG*, *mgtE*, *phoU*, and *aapM* code for proteins that regulate or directly facilitate the import and utilization of metabolic substrates. In addition, gene sequences of the metabolic enzymes *chlN* and *argF*, as well as a predicted sulfurtransferase, thioesterase, and amidase, each were under positive selection.

In contrast, positively selected genes in the Flavobacteriales were typically assigned to cell growth, repair, and replication functions (**Table 1**). A histidyl-tRNA synthetase gene (*hisS*), a DNA replication and repair gene (*recR)*, and two outer-membrane biogenesis and protein folding chaperones (*hlpA* and *ftsI*) were each under positive selection. The positively selected gene *purB* encodes the enzyme adenylosuccinate synthetase, which is involved in purine nucleotide biosynthesis during cellular replication and division.

## Discussion

The taxonomic structure of estuarine bacterioplankton communities is known to vary across salinity gradients (e.g., Crump et al., 1999; Smith et al., 2013) and between particle-attached and free-living fractions (e.g., D’Ambrosio et al., 2014; Zhang et al., 2016), but little is known about the physiological capabilities that results from this variability. Our study expands on this previous work by quantifying the functional capabilities and activities of taxa present under four distinct spatially separated and size-fractioned habitat conditions in the Yaquina Bay estuary.

Using amplicon sequencing of the v4 region of the 16S gene, we determined that microbial community-level taxonomic structure in Yaquina Bay was distinct between the upper and lower estuary margins and the free-living and particle-attached lifestyles in each location. Ordinations of these patterns in bacterial diversity based on 16S amplicon sequencing changed little using OTU-level or Order-level annotations and were each similar to taxonomic structure as determined by metagenome CDS sequence annotation (Supplementary Figure S9). Overall, we found patterns in the distributions of microbial populations that were similar to those in other temperate estuaries (e.g., Bouvier and del Giorgio, 2002; Ortmann and Santos, 2016).

### The Relative Abundance of Key Taxonomic Groups Distinguished the Four Sampled Habitats

Two highly abundant lineages, the Flavobacteriales and Rhodobacterales, contained 11 and 19 OTU-level populations that exhibited distinct habitat preferences. Both lineages had multiple OTUs enriched in each of the four habitats, resulting in no clear consensus of Order-level habitat preference. However, their two most abundant populations, a Cellulophaga (Flavobacteriales) and a Thalassobius (Rhodobacterales), were each relatively enriched in the upper estuary, leading to the significant influence of Flavobacteriales and Rhodobacterales in driving upper estuary communities apart from those inhabiting the lower estuary.

Pelagibacterales were also highly abundant in the estuary, but unlike Flavobacteriales and Rhodobacterales, the three Pelagibacterales OTUs exhibited strong habitat preferences, each being highly enriched in the planktonic fraction of the marine-influenced lower estuary margin and being most depleted in the particle-associated lifestyle of the upper estuary. These populations, which were all classified as SAR11 clade members, were most closely related to coastal (Ortmann and Santos, 2016), rather than brackish ecotypes (Smith et al., 2013), suggesting that they may have entered the estuary by tidal intrusion. This distribution of Pelagibacterales is consistent with the well-characterized planktonic lifestyle of SAR11 Subclade I (Giovannoni et al., 1990) and with its observed decrease in its relative abundance across another marine-estuary-river continuum (Ortmann and Santos, 2016).

Nitrosopumilales enrichment in the marine-influenced lower estuary (Hewson et al., 2014; Hugerth et al., 2015) and Burkholderiales in the riverine-influenced upper estuary (Bouvier and del Giorgio, 2002; Silveira et al., 2011) also corroborate previous findings of microbial population habitat range. Similarly, the high relative abundance of anaerobic or microaerophilic populations on suspended particulates is a common observation in estuarine environments (Crump et al., 1999; Waidner and Kirchman, 2007; Zhang et al., 2016), and may be the result of oxygen-depleted niches in particle biofilms (Dang et al., 2011), or suspension of colonized grains from the anoxic sediment by physical mixing (Crump and Baross, 1996; Baker et al., 2015). The specialist populations in particle-associated communities contributed to a significantly higher phylogenetic and metabolic diversity in the community associated with these samples, which is consistent with studies of suspended particulate material in other aquatic ecosystems (Smith et al., 2013; Dang and Lovell, 2015; Yung et al., 2016).

Overall, the results from community taxonomic structure analyses substantiate those from previous studies showing that both lateral gradients (Crump et al., 2004; Henriques et al., 2004) and filtration fraction of the water-column (Crump et al., 1999; Zhang et al., 2016) are significant predictors of microbial community structure in estuaries.

### Distinct Taxonomic Structure of Communities in Each Habitat Was Correlated With Distinct Functional Structure

We hypothesized that the distinct community taxonomic structure of each habitat would correspond to distinct functional structure, leading to differences in the capacity of each community to perform ecological processes such as carbon and nitrogen turnover. If supported, this habitat-specific ecological function may indicate how biogeochemical cycling in the estuarine water-column is spatially organized and provide a more complete understanding of how resources move through this ecosystem. If no significant functional differences were evident between habitats, it would suggest that communities encode functional redundancy despite their distinct taxonomic structure, leading to a uniform spatial distribution of biogeochemical cycling capacity and a dissociation between microbial population diversity and functional diversity (Allison and Martiny, 2008; Delgado-Baquerizo et al., 2016).

To determine community functional structure, we sequenced metagenomes of all samples and measured relative abundances of protein-coding gene families. Community functional structure was significantly correlated with taxonomic structure, leading to similar ordination shapes of OTU and KEGG relative abundance data. Lower estuary communities were enriched in central carbon metabolism functions (i.e., glycolysis and TCA cycle), while upper estuary communities were enriched in cell growth, transcription, and translation genes (Supplementary Figure S6). This result tracked with the distribution of microbial taxa with different trophic strategies: genomes of copiotrophic populations, such as those in the Rhodobacterales and Flavobacteriales lineages that were enriched in the upper estuary, typically encode relatively more transcriptional and ribosomal genes in their larger genomes, whereas oligotrophs, such as Pelagibacterales that were enriched in the lower estuary, often encode streamlined genomes with higher proportions of genes involved in resource transport and metabolism (Cottrell and Kirchman, 2016). Similar observations have been made across physico-chemical gradients in the brackish Baltic Sea, where key pathways and core metabolic processes were organized spatially by salinity gradients (Dupont et al., 2014).

Similarly, the relative enrichment of photosynthetic genes in particle-associated communities was related to the increased relative abundance of Cyanobacteria and chloroplast-like 16S v4 rRNA sequences in this size-fraction. In contrast, ABC transporters were overrepresented in free-living communities, which may be related to the enrichment of planktonic prokaryotes (e.g., SAR11, whose genome has a high density of transporter functions) in this water-column filtration fraction.

Enrichment for flagellar motility genes in the upper estuary differentiated the functional structure of communities in this margin from those in the lower estuary, which again may reflect the biased distribution of copiotrophic and oligotrophic lineages. The single flagellar motility gene that was overrepresented in the lower rather than upper estuary was an archaeal flagella subunit (K07332). This was expected considering that all 7 archaeal OTUs with significant habitat preferences were enriched in the marine-influenced lower estuary margin (OTUs 6, 30, 35, 42, 78, 161, 256).

### Communities Differed in Capacities for Carbon and Nitrogen Transformation

Based on these significant biases in the distributions of microbial community function across habitats, we hypothesized that microbe-mediated biogeochemical flux would be habitat-specific, leading to the spatial distribution of carbon- (C) and nitrogen- (N) cycling processes in the estuary. To test this hypothesis, we used metagenome and metaproteome data to estimate the relative abundance of marker genes and proteins involved in central C and N utilization pathways within each habitat community. Relative abundance ratios of these 31 functional markers were used to infer their capacity for catalyzing 10 major C- and N-cycling processes.

Out of the 31 metagenome marker genes, we detected 21 marker proteins in our samples, reflecting the difficulty in capturing total protein diversity from complex communities and the low recovery of protein fragments such as membrane-spanning domains (Williams and Cavicchioli, 2014). Regardless, all 10 C- and N-cycling processes we examined were represented by at least one marker protein, and the relative abundances of marker proteins generally tracked those of the marker genes that encoded them.

The two most abundant metabolic pathways, aerobic C respiration and N assimilation, had equal capacity across all habitat metagenomes and metaproteomes, suggesting that differences in taxonomic composition did not affect the capacity of the community to perform these functions. On the other hand, the relative abundance of markers for processes requiring specific environmental conditions was not conserved across habitats. For example, denitrification and fermentation genes and proteins were relatively more abundant in the particle-associated communities, especially in the lower estuary margin. The marker genes for these processes were predominately annotated to facultative or strictly anaerobic OTUs, which correspondingly were enriched in the particle-attached communities of both the upper and lower estuary, indicating that these metabolic specialists could be biogeochemically important members of the particle-associated communities (Jørgensen, 1977; Etcheber et al., 2007; Garnier et al., 2010; Zhang et al., 2016).

The relative abundance of encoded marker genes and expressed marker proteins for CO oxidation suggested that this process has the potential to contribute significantly to the C cycle of Yaquina Bay. CO oxidation capacity was highest in the upper estuary communities, and the majority (68%) of marker genes for this process were annotated to Roseobacter populations. Many OTUs in this clade were significantly enriched in the upper estuary (e.g., OTU 15, Litoreibacter; OTU 4, Thalassobius), and indeed Roseobacters have been proposed to be the major drivers of biotic CO oxidation in coastal ecosystems (Moran et al., 2004; Tolli et al., 2006). Estuarine CO is primarily produced through the photolysis or oxidation of particulate matter (Miller et al., 2002; Song et al., 2015), and the Yaquina Bay estuary and Yaquina river are known to be supersaturated in CO throughout the year (Butler et al., 1987). Thus, we hypothesize that a steady supply of CO from decaying particulate organic matter entering Yaquina Bay by riverine transport is consumed by Roseobacters in the upper estuary. Given the relative magnitude of this biogeochemical process based on marker gene and protein abundance, CO oxidation may be a key characteristic of upper estuary ecology, and changes in Roseobacter abundance or CO production may significantly change the flux of C from Yaquina Bay.

Unexpectedly, we did not detect KO marker genes for nitrification (K10944, K10945, or K10946), despite finding that the Nitrosopumilales lineage represented 0.6% of all 16S amplicons. When considering sample metagenomes, however, the relative abundance of genes annotated to these nitrifiers was an order of magnitude lower than 16S relative abundance (0.06%) and most recovered Nitrosopumilales genes were of housekeeping or unknown function (data not shown). This result indicates that the absence of nitrification markers in our samples may have been due to under-sampling of the genetic diversity in the system, rather true biological absence.

Importantly, our results highlight potential keystone organisms in the microbial food webs of the estuary surface water. For example, populations of Epsilon-proteobacteria (predominately of Sulfurospirillum) represented an ammonification marker gene abundance that was highly disproportional to their relative community abundance. Significant contribution to biogeochemical cycling by such populations in low-abundance lineages has been supported by studies examining rare biosphere activity (Campbell et al., 2011).

### Microbial Populations Appear to Use Varying Strategies to Succeed in the Estuarine Environment

To understand what characteristics may be allowing microorganisms to coexist across these four distinct habitats, we examined the pangenome and panproteome of the Flavobacteriales and Rhodobacterales lineages. The co-dominance of these lineages is a common observation in estuarine ecosystems (Dong et al., 2014; Colatriano et al., 2015), and they have been shown in experimental and observational studies to grow and acquire resources using different strategies (Alonso-Sáez and Gasol, 2007; Poretsky et al., 2010; Bryson et al., 2016, 2017). Thus, we hypothesized that their ability to coexist across the estuary may be due to the use of different strategies for growth and reproduction (Hibbing et al., 2010). This hypothesis is based on the phylogenetic limiting theory, which states that when populations minimize their overlap in life-strategy, deleterious interactions can be reduced between them, curtailing forces such as competitive exclusion that drive species with similar life-strategies out of a single habitat (Violle et al., 2011).

Our results supported the divergence in life-strategies of Flavobacteriales and Rhodobacterales, which we inferred based on the significantly different types of proteins expressed by each lineage at the time of sampling. Rhodobacterales populations dedicated a relatively larger part of their proteome to metabolic versatility and resource acquisition, while Flavobacteriales were enriched in transcription/translation and specialized starch transport functions. Similar patterns of protein-level expression and *de-novo* protein production have been recorded previously in these lineages using stable-isotope-probing experiments in Monterey Bay, in which Rhodobacterales accounted for the highest total amount of substrate assimilation (glucose, amino acids, peptides), while Flavobacteriales bloomed under all substrates but were significantly isotope-enriched only in a starch treatment (Bryson et al., 2017).

Interestingly, the types of functions under positive evolutionary selection in each lineage, which we determined using high-quality population genomes acquired from metagenome binning, appeared to be those that were related their respective life-strategies. This suggests that evolutionary selection may be a mechanism that maintains lineage life-strategies over time (Violle et al., 2011), allowing for the stable coexistence of Flavobacteriales and Rhodobacterales across the habitats we sampled. Indeed, the relationship between life-strategy and evolutionary selection has been proposed to contribute significantly to present-day observations of microbial function and community assembly (Luo and Moran, 2015). However, because our sampling was designed to focus on spatial rather than temporal investigation, it will be necessary to verify our interpretations over time (e.g., seasonal) or in other estuarine or coastal ecosystems.

Taken together, results from the investigation of Order-level pangenomes and panproteomes showed that the highly abundant and co-occurring Flavobacteriales and Rhodobacterales lineages were differentiated from one another not by broad functional groups that were encoded at the genome level, but by functions they expressed at the protein level. Results from the examination of evolutionary selection on genes from highly resolved, population-level genomes in each lineage showed that positive selection on OGs encoded in Flavobacteriales and Rhodobacterales appeared to be acting on gene sequences with the types of functions that confer the life-strategy of both groups (i.e., genes in **Table 1** were biased toward growth and replication functions for Flavobacteriales and metabolism-related functions in Rhodobacterales). This is the first such example of a connection between life-strategy and evolutionary selection in estuarine microorganisms, though a link between functions under positive selection and functions that are important in defining the life-strategy of microbial populations has been observed previously, including studies of deep sea bacteria (Campanaro et al., 2008) and extremophilic archaea (Gunbin et al., 2009).

## Data Availability

All 16S-amplicon and metagenome sequences are available from NCBI (BioSample SAMN04917373, BioProject PRJNA320136, short read archive SRS1422236 with accession numbers SRX1738750-90 for 16S-amplicon reads and SRX1738728-32 for metagenome reads). The metaproteome protein data are available from the PRIDE archive under ID PXD008093.

## Author Contributions

BK, SB, XM, RH, CP, and RM designed the experiments. BK, SB, XM, and RM coordinated and carried out the sampling procedures. BK, ZL, RH, CP, and RM performed the analytical procedures and acquired data. All authors contributed to discussion of results. ZL, RH, CP, and RM contributed reagents, materials, and technology. BK and RM designed and prepared figures and tables and drafted the original manuscript. All authors contributed critical revisions during the editing process, and all authors approved the final manuscript draft for submission.

## Conflict of Interest Statement

The authors declare that the research was conducted in the absence of any commercial or financial relationships that could be construed as a potential conflict of interest.
