# Supplementary material for: Microbial Community Structure–Function Relationships in Yaquina Bay Estuary Reveal Spatially Distinct Carbon and Nitrogen Cycling Capacities
Source: Front Microbiol. 2018 Jun 14;9:1282. doi: 10.3389/fmicb.2018.01282 (PMC6010575; doi:10.3389/fmicb.2018.01282)
Supplement: Supplementary file 2 [file Presentation_1.PDF]

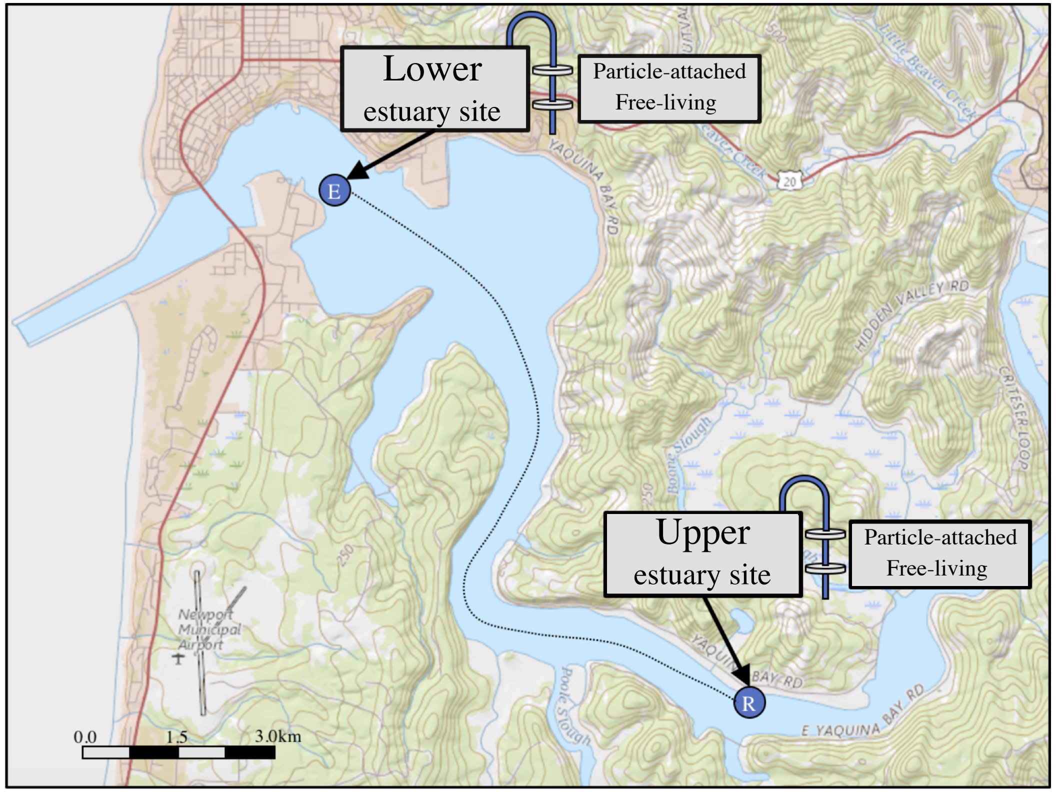


***Fig. S1**. Microbial communities were sampled from surface water in four estuary habitats*. Upper and lower estuary sites were ~8.5 km of over-water distance apart and size-fractions were defined as particulate-associated (>3$\mu$m) and planktonic (0.22-3$\mu$m) cells with in-line peristaltic filtration. Map produced with ArcGIS Desktop (Redlands, CA, USA).

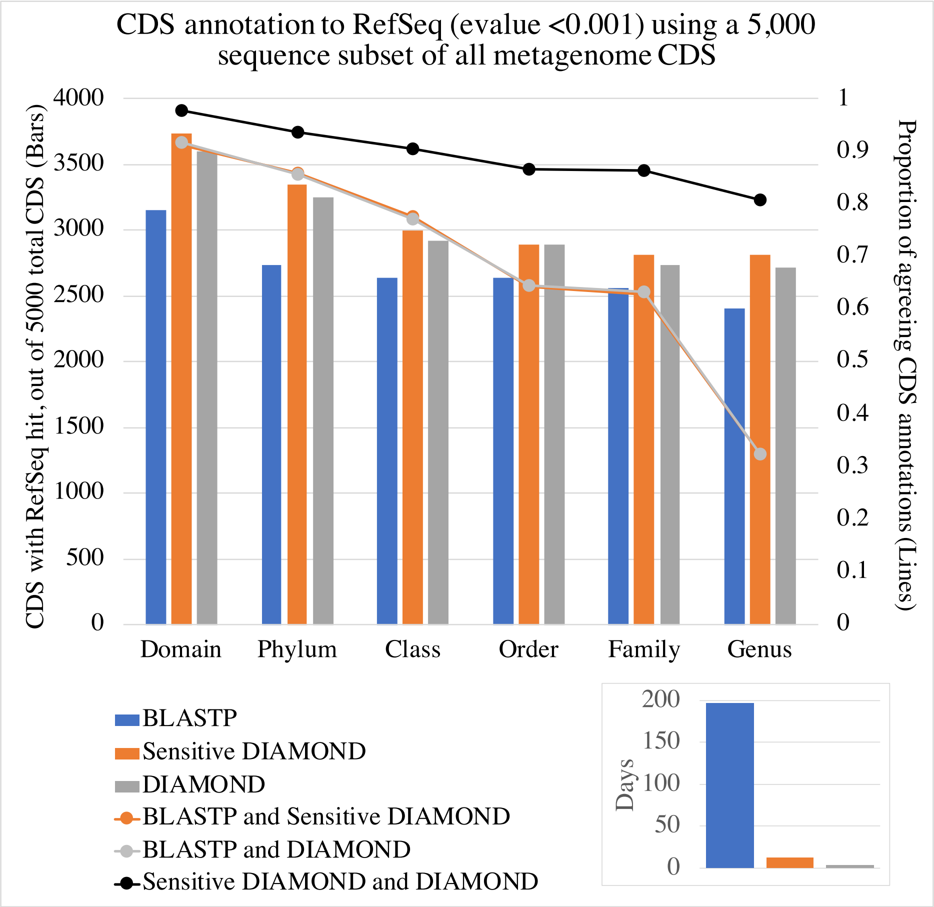


***Fig. S2.*** *Speed and annotation tests were performed using the BLASTP, DIAMOND BLASTP, and DIAMOND BLASTP (Sensistive mode) programs in order to determine the algorithm best suited for annotation of metagenome CDS during carbon and nutrient cycling marker gene analyses*. Based on this test, it was determined that DIAMOND BLASTP was the most time-efficient algorithm and retained high annotation agreement with Sensitive mode at the Class level (91%), which was used in analyses (BLASTP was determined to be too slow to annotate the metagenome CDS dataset - see inset). The main panel shows bars representing number of CDS with annotations (not unclassified) at each taxonomic level using each search algorithm (left axis), and lines, representing, of those CDS annotated, the proportion of annotations at each taxonomic level that agreed between methods. The inset shows the estimated number of days (24 hours) it would take to complete the annotation of all metagenome CDS with each method. 5,000 CDS were chosen at random from the full metagenome dataset for the speed and annotation tests.

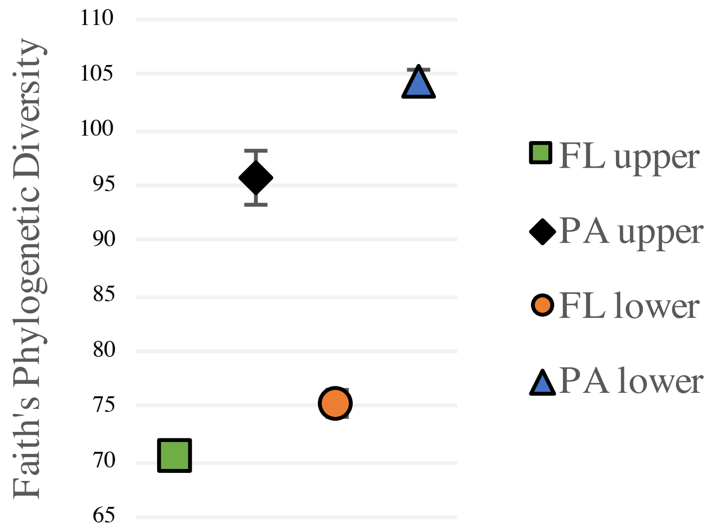


| Comparison | p-value |
|---|---|
| All: Upper_vs_Lower | 0.45204322 |
| All: FL_vs_PA | 9.9913E-06 |
| PA only: Upper_vs_Lower | 0.08463409 |
| FL only: Upper_vs_Lower | 0.07563017 |
| Upper only: FL_vs_PA | 0.00630402 |
| Lower only: FL_vs_PA | 0.00021224 |

***Fig. S3**. Phylogenetic diversity comparisons between habitats*. Table shows pairwise Welch's t-test p-values, with significance set at p = 0.05. FL = Free-living; PA = Particle-attached. Error bars are based on standard errors of three biological triplicates of 16S amplicon libraries.

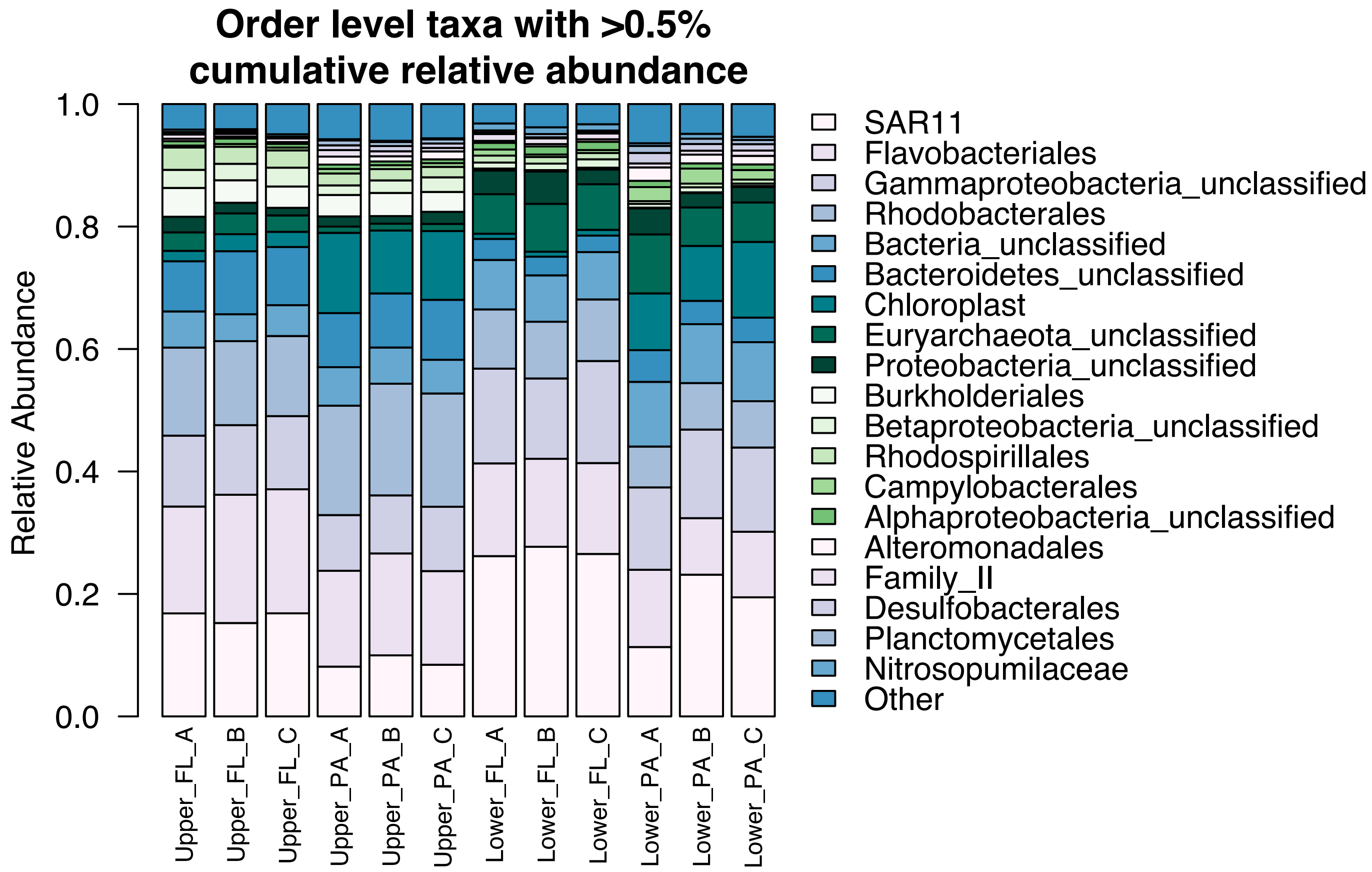


***Fig. S4***. *Taxonomic community structure across Habitats*. Relative abundance of OTUs grouped at Order-level taxonomy are shown for each replicate 16S rRNA library. OTUs with <0.5% relative abundance are shown as “Other”. FL = Free-living; PA = Particle-attached.

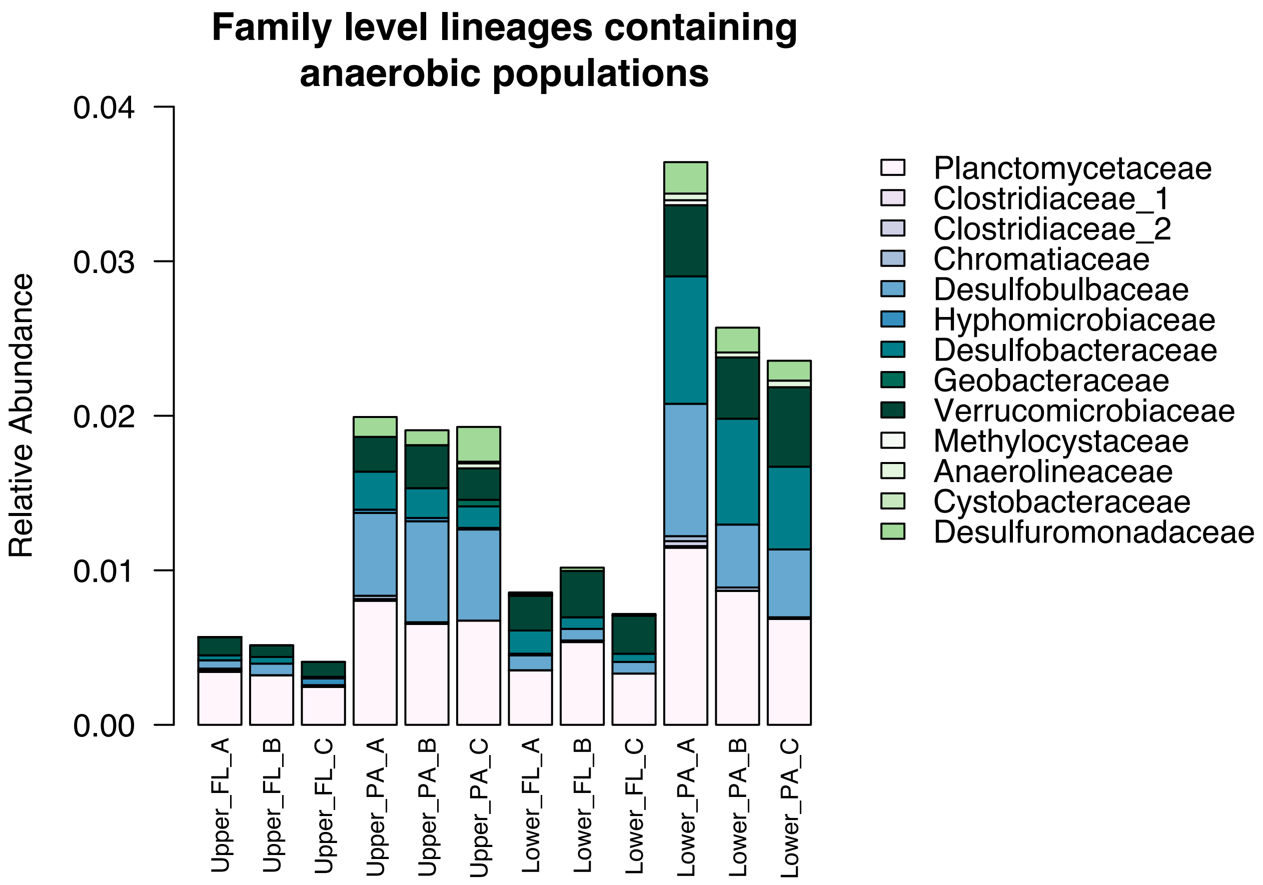


***Fig. S5***. *Cumulative relative abundance in the 16S dataset of Family-level lineages that contained putative strict or facultative anaerobic OTUs*. FL = Free-living; PA = Particle-attached.

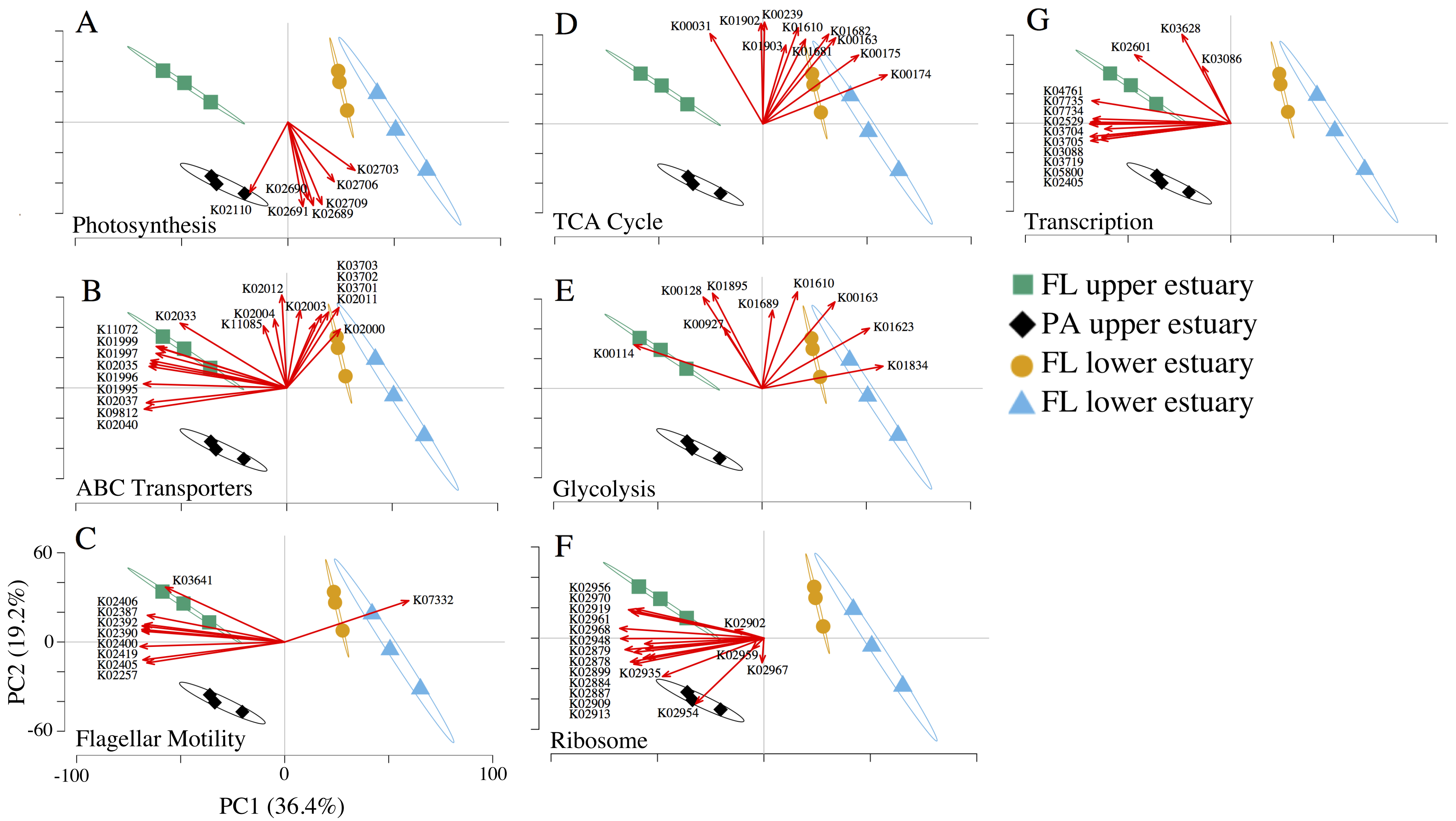


***Fig. S6***. *PCA of functional (metagenome KOs) influence on Habitat clustering*. Each panel shows the same PCA KO plot as in Fig. 2, with axes drawn on only the lower-left plot for clarity. Bi-plots show all significant habitat-enriched KO numbers (from LEfSe analysis) for the KO pathways of (A) Photosynthesis, (B) ABC transporters, (C) Flagellar motility, (D) TCA Cycle, (E) Glycolysis, (F) Ribosome, and (G) Transcription. Descriptions of the KO numbers plotted can be found in Table S6. FL = Free-living; PA = Particle-attached.

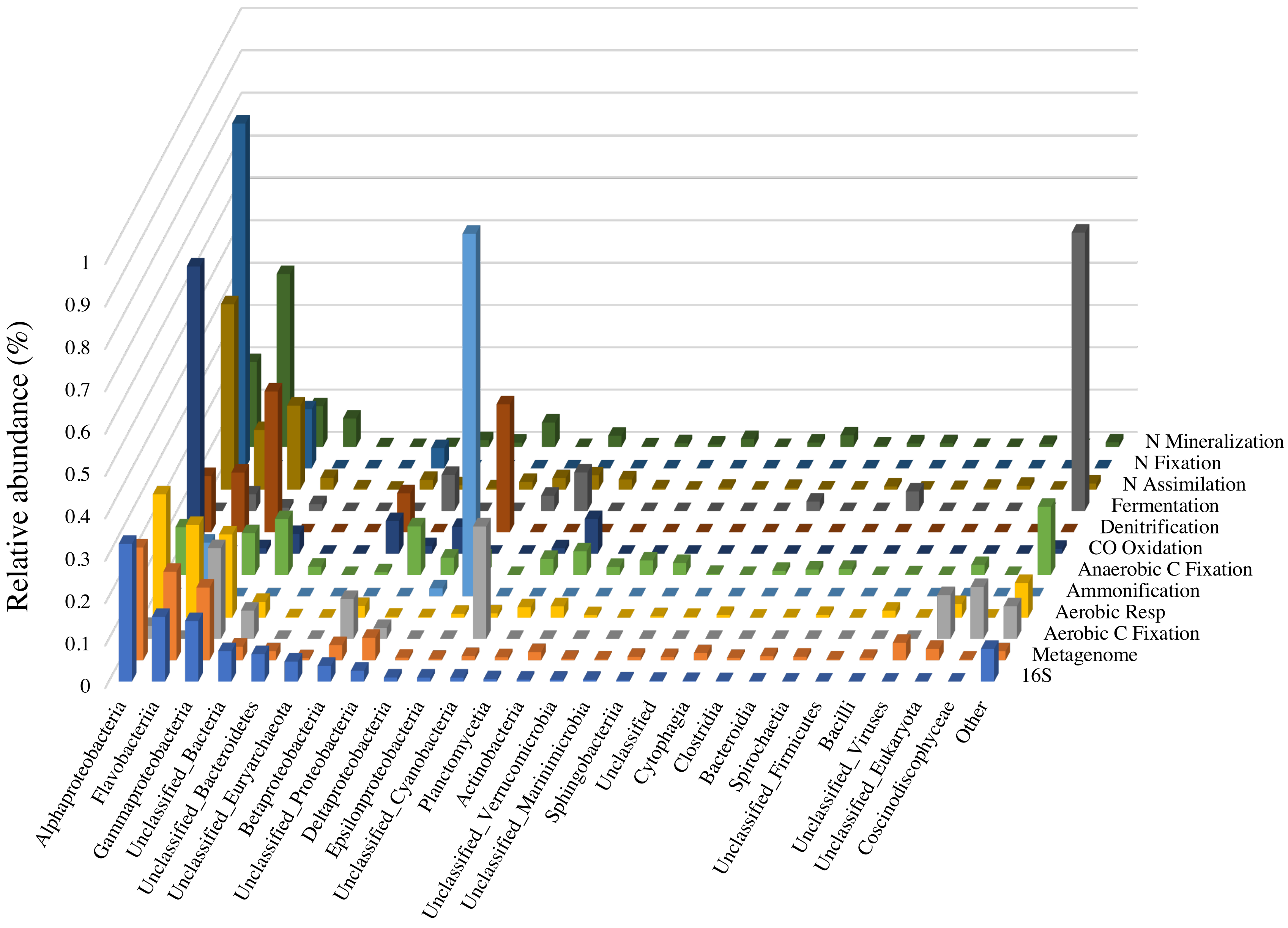


***Fig. S7**. Representation of taxonomic groups (cumulative across all samples) in each elemental cycling process*. Taxa are arranged by rank-abundance in the 16S dataset (closest z-axis row). Taxon relative abundance in the metagenome CDS dataset and each of the metabolic marker gene sets is shown as additional rows on the z-axis.

Rhodobacterales

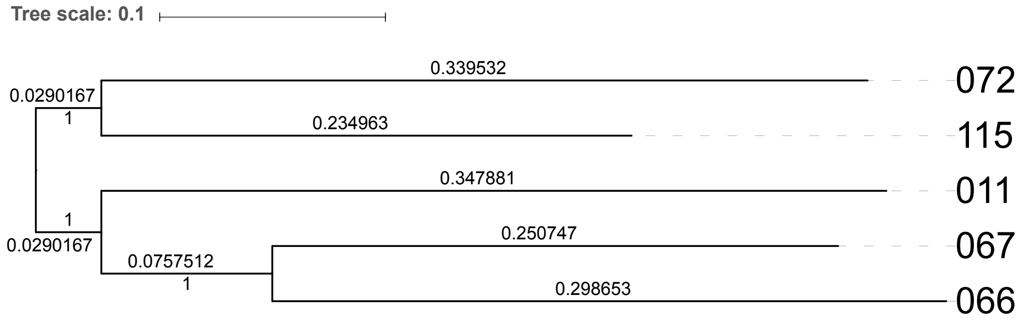


Flavobacteriales

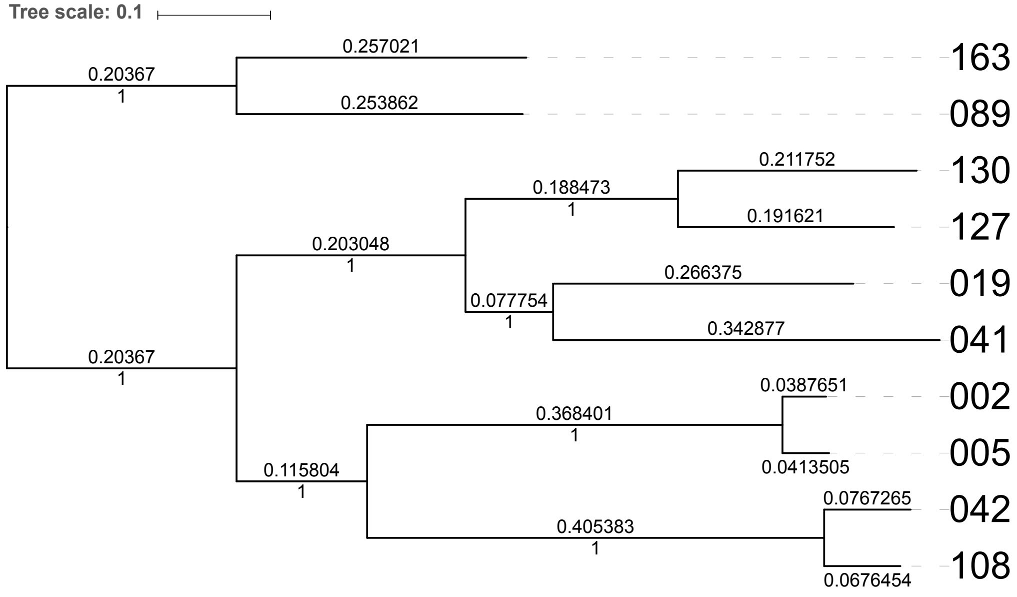


***Fig. S8***. *Phylogenomic relationships of binned populations within each lineage*. The concatenated tree is based on the core OG amino acid multiple sequence alignments using RAxML (Number of OGs: Rhodobacterales, 138; Flavobacteriales, 73). Scale bars measure percent divergence in amino acid sequence identity. Bootstrap values are shown below each branch. Leaf labels represent populations whose taxonomic identity and binning statistics can be found in Supplemental Table 7.

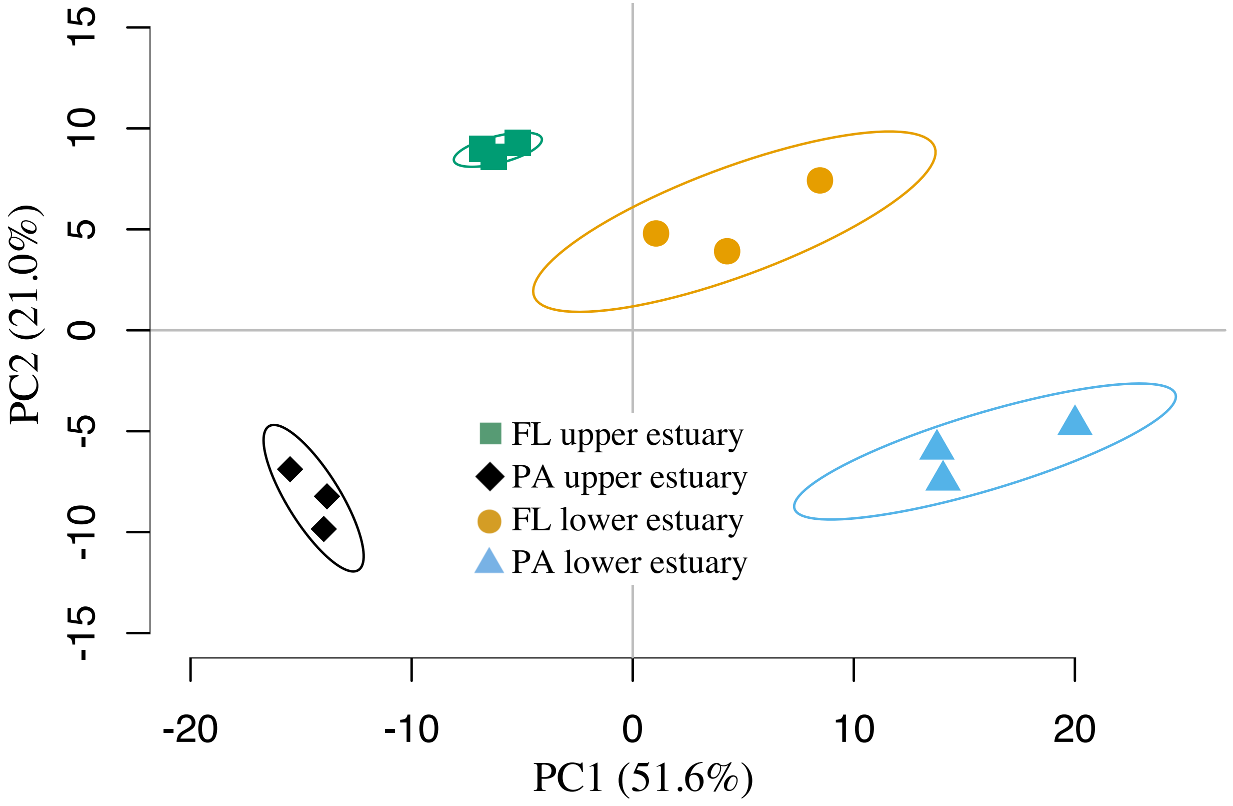


***Fig. S9**. Euclidean distance PCA of metagenome CDS abundance collapsed to Order-level taxonomic groups*. Sample triplicates per habitat are plotted based on the RPKG abundance of CDS annotated to a taxonomic group and collapsed at the Order level. Ellipses represent 95% confidence intervals for centroid positions. FL = free-living; PA = particle-attached.
